# An Efficient Synthesis of Novel Bioactive Thiazolyl-Phthalazinediones under Ultrasound Irradiation

**DOI:** 10.3390/molecules22020319

**Published:** 2017-02-18

**Authors:** Fatma S. Elsharabasy, Sobhi M. Gomha, Thoraya A. Farghaly, Heba S. A. Elzahabi

**Affiliations:** 1 Department of Chemistry of Natural and Microbial Products, National Research Center, Dokki 12622, Egypt; elsharabasy2000@gmail.com; 2 Collage of Science and Humanities, Sattam bin Abdul Aziz University, Alkharj City 11942, Saudi Arabia; 3 Department of Chemistry, Faculty of Science, Cairo University, Giza 12613, Egypt; s.m.gomha@gmail.com; 4 Department of Chemistry, Faculty of Applied Science, UmmAl-Qura University, Makkah Almukkarramah 21514, Saudi Arabia; 5 Department of Pharmaceutical Chemistry, Faculty of Pharmacy (Girls), AlAzhar University, Cairo1 1754, Egypt; helzahabi@yahoo.com

**Keywords:** thiazolylphthalazinediones, hydrazonoyl chlorides, ultrasound irradiation, antimicrobial activity

## Abstract

Novel 2-thiazolylphthalazine derivatives were efficiently synthesized under ultrasound irradiation, resulting in high yields and short reaction times after optimization of the reaction conditions. All prepared compounds were fully characterized using spectroscopic methods. They were screened for their antimicrobial activity against Gram-positive and Gram-negative bacteria as well as for antifungal activity. The antimicrobial activity profile of the tested compounds showed some promising results. The potent activity of compounds **4d**, **7b** (117% zone inhibition) and **7c** (105% zone inhibition) against *Salmonella sp.*, exceeding that of the reference drug *Gentamycin* is particularly noteworthy. In general, the newly synthesized thiazolylphthalazine derivatives showed higher antimicrobial activity against the tested Gram-negative bacteria than against Gram-positive bacteria and fungi.

## 1. Introduction

**Figure 1:**
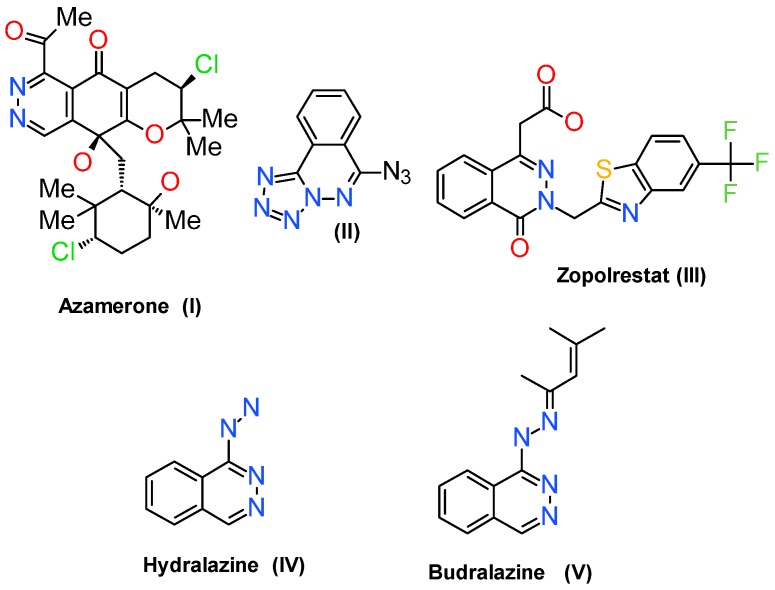
Natural products and marketing drugs containing phthalazine moiety.

There are only two naturally occurring phthalazine derivatives, namely azamerone (**I**, Figure 1), which was isolated in 2006 from amarine-derived *Streptomyces* species [1], and 6-azidotetrazolo[5,1-*a*]phthalazine (**II**, Figure 1), which was isolated in 1985 from *Gymnodiniumbreve*, a toxic red-tide dinoflagellate [2]. Some phthalazine derivatives have been reported to possess anticonvulsant [3], antitumor [4,5,6], anti-inflammatory [7], and antidiabetic and vasorelaxant activities [8]. Phthalazines have also shown interesting vasodialatory and antihypertensive properties [9]. It is important to point out that phthalazine moiety is at the core of many commercial drugs. For example, zopolrestat (**III**, Figure 1) is a drug used to decrease blood glucose levels, while hydralazine (**IV**, Figure 1) and budralazine (**V**, Figure 1) display vasodilating effects.

One of the most pivotal contributions of phthalazine derivatives is their antimicrobial activity. According to the literature, the most common substitution pattern of the target phthalazine skeleton is 1-,2- and 4- on the diazine part of the bicyclic system [10,11,12]. One extensive study illustrated the remarkable antifungal activity of 4-substituted-2-methylphthalazines, which as a result could be considered good lead compounds [13].

Moreover, the bicyclic phthalazine skeleton has been proven to display appreciable broad spectrum antimicrobial activity against Gram-positive and Gram-negative bacteria as well as fungi [14]. In a recent study [15], various phthalazine derivatives bearing aliphatic, aryl, and heteroaryl side chains at the C2 position of the phthalazine skeleton exhibited potent antimicrobial effects. In addition, some sugar-based phthalazine compounds also act as broad spectrum antimicrobial agents [15].

**Figure 2:**
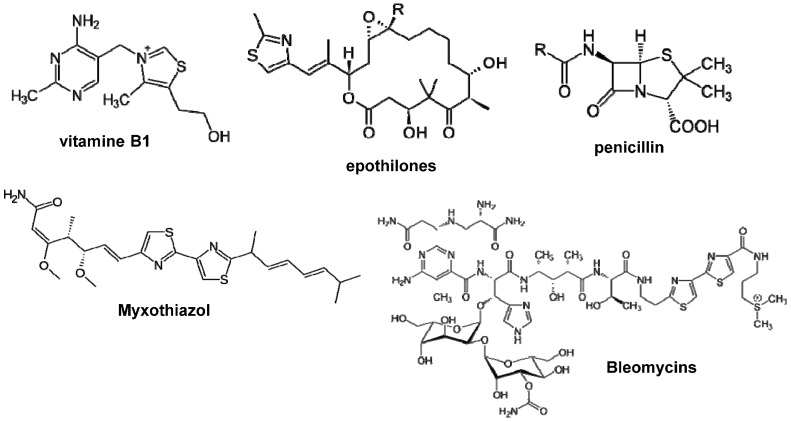
The structures of vitamin B1, the epothilones, penicillins, myxothiazol and bleomycin.

Another important heterocyclic ring is the thiazole ring, which is an interesting building block found in a range of natural products and part of many potent biologically active molecules such as vitamin B1, the epothilones, penicillins, myxothiazol and bleomycin (Figure 2).

The value of the thiazolyl moiety as an antimicrobial agent has been reported in many studies. Some thiazolyl-based scaffolds with alkyl chains or aromatic rings at C5 also display potent antimicrobial activity [16]. 

Ultrasound-promoted synthesis has attracted much attention during the past few decades as a clean and useful procedure protocol in organic synthesis compared with traditional methods [17,18,19]. This is due to the method’s cavitation process, which causes the formation, expansion, and collapse of a huge number of bubbles within short time, inducing rapid and violent implosions that create hot spots with very high local temperatures and pressures that which can be thought of as microreactors in which the sound energy is converted into a chemically useful form [17]. Considering the preceding reports, and in continuation of our work on the synthesis of bioactive compounds [20,21,22,23,24,25,26] and considering the worldwide antibiotic resistance crisis [27], we report herein our synthesis of a new series of thiazolylphthalazines using ultrasound irradiation with the aim of decreasing the reaction times and increasing the yields of the reaction products. In the synthesis described in this work we have adopted a molecular hybridization approach for the purpose of designing novel and more potent antimicrobial agents. To this end the target nuclei are hybrid thiazolyl scaffold-based phthalazine moieties, in which the phthalazine system is linked to different substituted thiazolyl nuclei at the C2 atom.

## 2. Results and Discussion

### 2.1. Synthesis

In continuation of our previous work on the synthesis of bioactive heterocyclic compounds under mild conditions [28,29,30], we report here simple and efficient procedures for the synthesis of some novel thiazolylphthalazinedione compounds via the reaction of 1,4-dioxo-3,4-dihydro-phthalazine-2(1*H*)-carbothioamide (**1**) with different halogenated compounds under ultrasound irradiation. In order to optimize the reaction conditions, the reaction between compound **1** and hydrazonoyl chloride **2a** was carried out using different solvents at two different temperatures (room temperature and 50 °C) under ultrasound irradiation (Table 1). In those experiments, we observed that the reactions were solvent dependent and, as shown in Table 1, the most suitable conditions involved ethanol as a solvent at 50 °C.

In addition, we compare the efficiency of ultrasound conditions with conventional heating, considering there action yield and time. Table 2 shows that our method is simpler, more efficient, and less time consuming for the synthesis of thiazolylphthalazine derivatives.

**Scheme 1:**
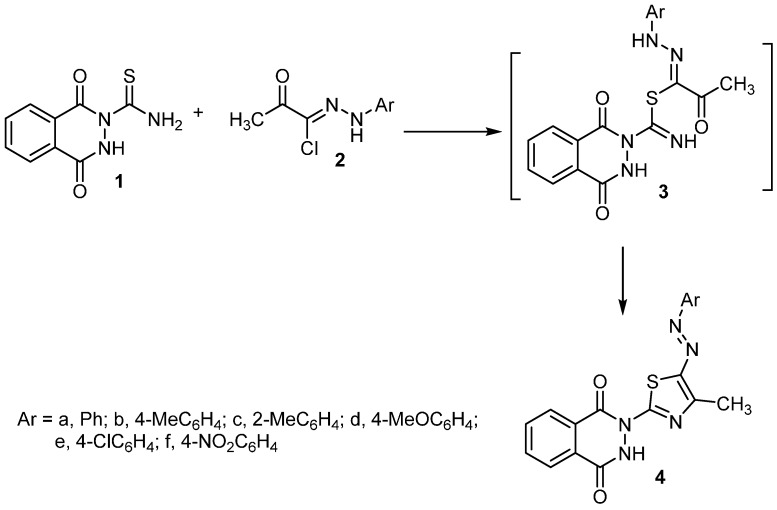
Synthesis of the thiazole derivatives **4**.

The reaction between 1,4-dioxo-3,4-dihydrophthalazine-2(1*H*)-carbothioamide (**1**) and acetyl hydrazonoyl chloride derivatives **2** in ethanol, in the presence of a base catalyst under optimized conditions, yielded only one isolated product (Scheme 1). The spectroscopic information confirmed the formation of products **4**, likely produced via intermediates **3** by elimination of a water molecule (Scheme 1). For example, the ^1^H-NMR spectra of compounds **4a**–**f**, which exhibited singlet signals at *δ* 2.55–2.60 ppm (CH_3_) and one D_2_O exchangeable peak at *δ* 9.18–10.71 ppm corresponding to the phthalazine-NH.

**Scheme 2:**
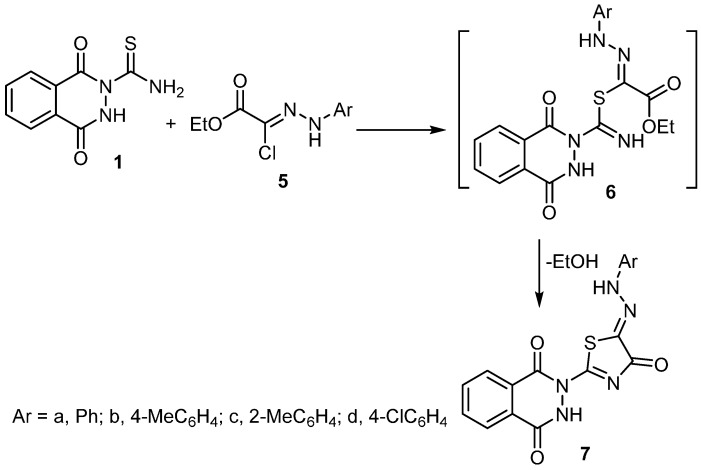
Synthesis of thiazole derivatives **7**.

**Scheme 3:**
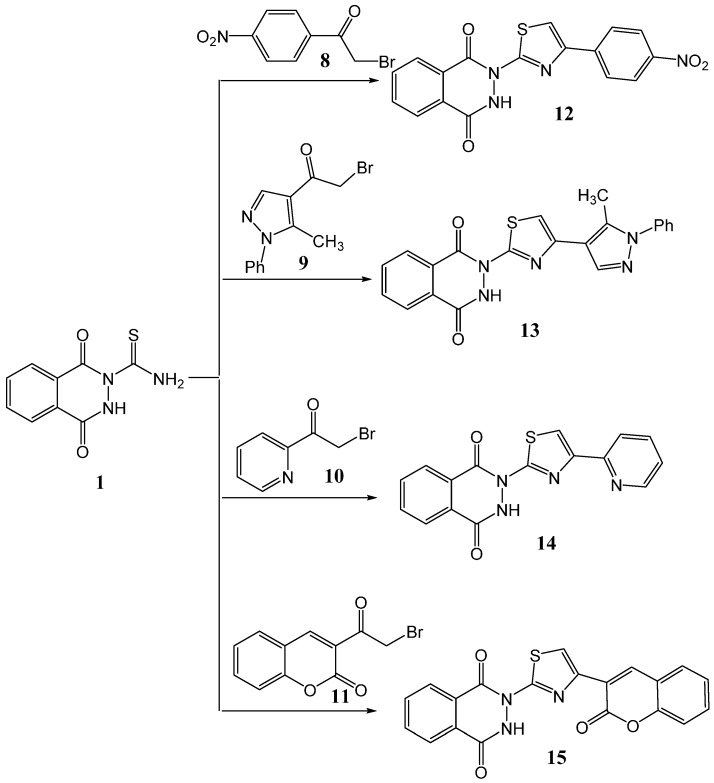
Synthesis of the thiazole derivatives (**12**–**15**).

In an identical way, when carbothioamide **1** was allowed to react with the ethyl (*N*-arylhydrazono)-chloroacetates **5** under the same reaction conditions, it yielded in each case a single product, namely, the 2-(4-oxo-5-(2-arylhydrazono)-4,5-dihydrothiazol-2-yl)-2,3-dihydrophthalazine-1,4-dione **7** (Scheme 2). The structure of compounds **7a**–**d** was proved using spectral data and elemental analyses (see Section 3). The carbothioicacidamide **1** seemed to be useful synthon for the synthesis of different heterocyclic compounds. Thus, the reaction of compound **1** with halogenated ketones **8**–**11** afforded new thiazole derivatives **12**–**15** (Scheme 3). These reactions were carried out under ultrasound irradiation and gave excellent yield in short reaction times (Table 3). The structure of compounds **12**–**15** was determined using spectroscopic data. For example, the ^1^H-NMR spectrum of compound **15** showed the following signals: 7.37–8.65 (m, 8H, Ar-H), 8.36 (s, 1H, coumarine-H4), 8.64 (s, 1H, thiazole-H5), 8.87 (s, br, 1H, NH, D_2_O-exchangeable). The mass spectrum of the thiazolylphthalazine derivative **13** revealed the existence of a molecular ion peak at *m*/*z* = 401. Also, the infrared spectra of thiazoles **12**–**15** were free from any absorption bands characteristic of the NH_2_ group.

**Scheme 4:**
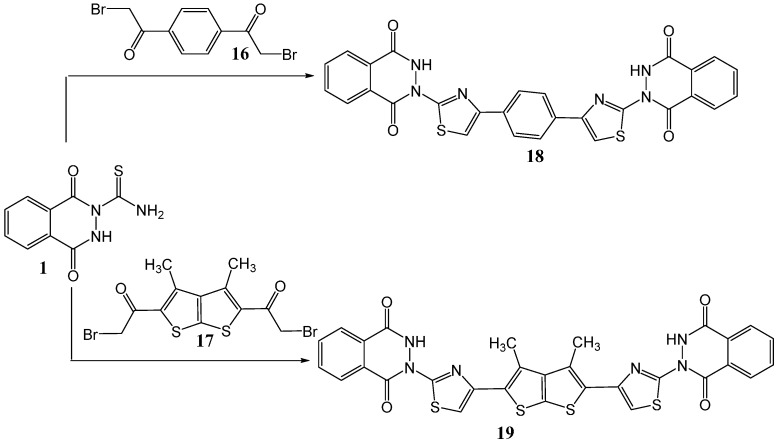
Synthesis of the *bis*-thiazole derivatives **18** and **19**.

From literature reports [31,32,33] we found that compounds bearing more than one thiazole ring unit also exhibit good biological activities. For example, myxothiazol (Figure 2) is an inhibitor of the mitochondrial cytochrome bc1 complex, and bleomycins is an anti-cancer agent, containing 2,4′-*bis-*thiazole system. From the above findings, we thought it is useful to synthesize a heterocyclic ring system carrying *bis*-thiazole moiety incorporated with a phthalazine ring. This aim was achieved via the reaction of *bis*-bromoketones **16** and **17** with the carbothioic acid amide **1**, also under ultrasound irradiation to afford a good yield of the *bis*-thiazoles **18** and **19** (Scheme 4, Table 3). The structure of compounds **18** and **19** was proved using spectral data and elemental analyses (Section 3). 

### 2.2. Antimicrobial Activity 

The protocol of antimicrobial screening adopted an agar diffusion technique [34] at the Regional Center of Mycology and Biotechnology, Al-Azhar University, Cairo, Egypt. All synthesized compounds were screened for their antifungal and antibacterial activities (gram-positive and gram-negative) at concentrations of 5 mg/mL. Amphotericine *B*, ampicillin and gentamycin were used as standard antifungal and antibacterial agents (gram-positive and gram-negative) respectively. The tested fungi were *Aspergillus fumigates* and *Candida albicans*. Tested gram-positive bacteria were *Staphylococcus aureus* and *Bacillus subtilis*, while gram-negative were *Salmonella sp.* and *Escherichia coli.* Susceptibilities of microbial isolates to the test compounds were recorded by measuring the average diameter of bacterial growth inhibition zones surrounding the well (in millimeters) compared to those surrounding the reference drugs. The obtained data indicated variable responses of cultured microorganisms towards the test compounds **1**, **4a**–**f**, **7a**–**d**, **12**–**15**, **18**, and **19**. So, diverse inhibitory zones ranges (0 to 21 mm) resulted, compared to the reference drugs. The inhibitory zone profile of the microbial isolates is shown in Table 4. 

#### 2.2.1. Antibacterial Activity

The performance of the test compounds against Gram-positive *S. aureus* did not exceed that of the ampicillin used as a reference standard. Noticeably, compounds **4b** and **18** exerted moderate activity (69% and 65% inhibition zones, respectively) compared to ampicillin. Compounds **4f** and **15** exerted a 60% inhibition zone ratio compared to ampicillin. On the other hand, **1**, **7a**–**d**, **12**, and **13** were devoid of any activities against *S. aureus. Bacillus subtilis* exhibited higher resistance rates towards the test compounds. Compound **4b** was the only one that exhibited a 50% inhibition zone ratio compared to ampicillin. The activity profile against Gram-negative bacteria indicated remarkable activity against *Salmonella sp*. Among the test compounds, four pthalazine derivatives exceeded the activity of gentamycin as a reference standard. Thus, the relative inhibition zone of **7c** was 105% and those of **4d** and **7b** were 117%, compared to that of gentamycin. Compounds **4a**, **4b**, **4f**, and **18** were equipotent, exerting high activity with an 88% inhibition zone ratio. The activities of **4c** and **14** were appreciable, with 82% and 70% inhibition zone ratios, respectively (Table 4). However, the profiles against the second Gram-negative bacterium, *Escherichia coli*, were quite different. *Escherichia coli* cultures displayed a higher resistance rate than *Salmonella sp.* Only compounds **4b**, **14** (84% inhibition zone each), and **18** (78% inhibition zone) exerted high activity rates compared to gentamycin.

#### 2.2.2. Antifungal Activity

The recorded data reflected weaker antifungal profiles for the test compounds **1**,**4a**–**f**, **7a**–**d**, **12**–**15**, **18**, and **19** than as antibacterial agents. *Aspergillus fumigates* exhibited a high resistance rate, as only compounds **14**, **15**, and **18** showed moderate activity ranging from 60%–69% inhibition zone ratios compared to Amphotericin B. On the other hand, compounds **1**,**4b**, **4c**, **4f**, **7b**, **7c**, and **19** exhibited mild-to-weak activity, showing an inhibition zone percentage range of 34%–52%. The rest of the test compounds were in active.

In contrast, *Candida albicans* showed greater sensitivity toward compound **18**, which exhibited high antifungal activity with an 84% inhibition zone rate compared to Amphotericin B. Compounds **4c**, **4f**, and **19** showed moderate activity with a 60% inhibition zone, while others showed poor-to-negative effects. In conclusion, the antimicrobial profile of the test compound included some promising results. Noticeably, compound **18** demonstrated abroad antimicrobial spectrum. It exhibited high antibacterial activity against *Salmonella sp.* (88% potency), with concomitant good antifungal activity against *Candida albicans* (84% potency). Also, it exerted moderate activities against *Escherichia coli* (78% potency) and *S. aureus* (65% potency). Compound **4b** was potent against *Salmonella sp.* (88% potency) with concomitant good activity against *Escherichia coli* (84% potency) and moderate activity against *S. aureus* (69% potency). In particular, the remarkable activity of **4f** against *Salmonella sp*. (88% potency) could not be ignored. An interesting issue is the potent activity of **4d**, **7b** (117% zone inhibition), and **7c** (105% zone inhibition) against *Salmonella sp.*, exceeding that of the reference drug gentamycin. Generally, the profiles of the newly synthesized thiazolylphthalazine derivatives exhibited higher antimicrobial activity against the tested Gram-negative bacteria than Gram-positive ones andfungi.Thetestcompoundsshouldbesubjectedtofurtherinvestigationto identify selective lead compounds.

#### 2.2.3. Structure-Activity Relationships (SAR)

**Figure 3:**
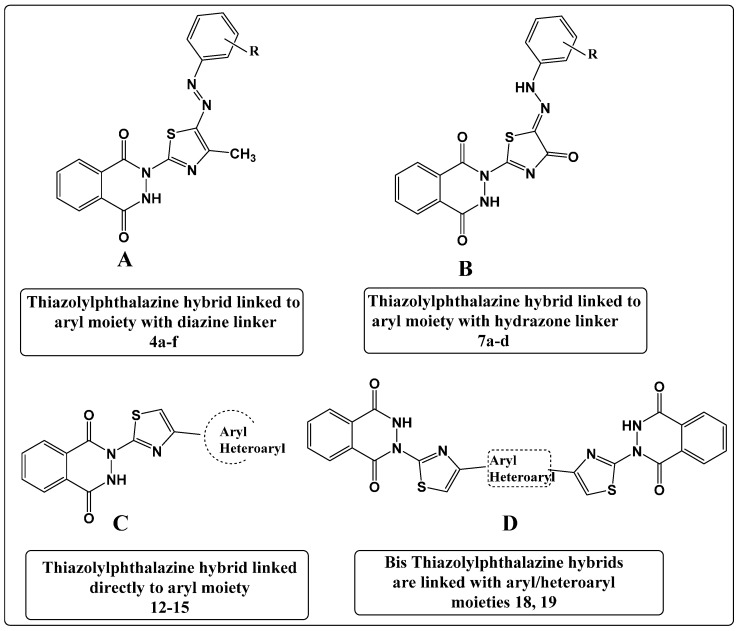
Structural profile map of the test compounds.

Adopting the hybridization approach, a new series of novel thiazolylphthalazine hybrids was synthesized. The test compounds are mapped into four structure profiles: A (**4a**–**f**), B (**7a**–**d**)*,* C (**12**–**15**), and D (**18**,**19**) employing phthalazinecarbothioamide **1** as a starting precursor (Figure 3). The successful synthesis of effective antimicrobial agents was demonstrated by the significant effects against gram-negative bacteria (*Salmonella sp.*) seen in the screening. The recorded data confirmed compounds **4b**, **4d**, and **4f** as the most potent derivatives in group A against *Salmonella sp.* compared to gentamycin. Precursor **1** exerted high antimicrobial activity (94% inhibition zone) that increased remarkably to a 117% inhibition zone in compound **4d**. This result was resulted after incorporating the non-cyclic thiamido group in **1** in to the heterocyclic thiazolyl moiety in **4d**. Also, the SAR study illustrated the positive effect of a *p*-methoxyphenyl group linked to the diazine moiety at C5 of the thiazole ring. On the other hand, **4a**, **4b**, and **4f** possess similar structures, differing in the substitution the *p*-OCH_3_ moiety by a *p*-H, *p*-CH_3_ or *p*-NO_2_, respectively, which each resulted in 88% activity, so the SAR comparison demonstrated that incorporation of a hydrophilic electron-donating group in the *para* position, for example the *p*-OCH_3_ group in **4d**, greatly increased the antimicrobial activity. Also, switching from *p*-CH_3_ in **4b** to *o-*CH_3_ in **4c** slightly decreased the inhibition zone activity from 88% to 82%. Finally, the existence of a *p*-chloro group in **4e** abruptly decreased activity against *Salmonella sp*. To an inhibition zone of only 52%. Regarding the second group, B (compounds **7a**–**d**), Gram-negative bacteria, for example *Salmonella sp*., were the most sensitive organisms to this group. Compounds **7b** and **7c** exerted potent activity, with 117% and 105% inhibition zones, respectively. Their structural profiles included a hydrazone moiety as a linker. Noticeably, when the *p*-CH_3_ group in **7b** was switched to *o-*CH_3_ in **7c**, a concomitant slight decrease in activity was noted. Similarly, switching the CH_3_ moiety in group A between **4b** and **4c** slightly affected their activity.

Thus, it seemed that the steric effect of the phenyl group in **4b**,**c**, and **7b**,**c** did not play agreat role in the antimicrobial activity against *Salmonella sp.* Again, *p*-chlorosubstitution in **7d** completely abolished the activity and the plain phenyl group in **8a** as good. Group C (compounds **12**–**15**) proved the vital role of either diazine or hydrazone linkers. Noticeably, direct linking between the thiazolylpthalazine hybrid and the aryl/heteroaryl moieties exerted a negative impact on the antimicrobial activity. Compound **15** in group C showed a slight improvement of activity, which could be attributed to the existence of the coumarin nucleus [35]. Further, the bimolecular system in group D (compounds **18** and **19**) possesses appreciable antimicrobial activity. Compound **18**, bearing a phenyl group linker between the bimolecular thiazolylphthalazine hybrids, exhibited better activity. It selectively inhibited the growth of *Candida albicans* and *Salmonella sp.* (84% and 88% inhibition zones, respectively). Among the four groups, **18** was the only compound that showed high antifungal activity against *Candida albicans*.

## 3. Materials and Methods

### 3.1. General Experimental Procedures

Melting points were measured with an IA 9000-series digital melting-point apparatus (Bibby Sci. Lim. Stone, Staffordshire, UK). IR spectra were recorded in potassium bromide discs on FTIR 8101 PC infrared spectrophotometers (Shimadzu, Tokyo, Japan). NMR spectra were recorded on a Mercury VX-300 NMR spectrometer (Varian, Inc., Karlsruhe, Germany) operating at 300 MHz (^1^H-NMR) and run in deuterated dimethylsulfoxide (DMSO-*d*_6_). Chemical shifts were related to that of the solvent. Mass spectra were recorded on a Shimadzu GCeMS-QP1000 EX mass spectrometer (Tokyo, Japan) at 70 eV. Elemental analyses were measured using an ElementarVario LIII CHNS analyzer (elementar Analysensysteme GmbH, Hanau, Germany). Ultrasonication was carried out in an ElmaRD-7700 apparatus (Singen, Germany). The electric supply was 230 V, A.C. 50 Hz, 1 phase; the ultrasonic frequency was 36 KHz and the ultrasonic power 100 W. 1,4-Dioxo-3,4-dihydrophthalazine-2(1*H*)-carbothioamide (*1*) [36] and hydrazonoyl halides 2 and 5 [37] were prepared as previously reported in the respective literature.

### 3.2. Synthesis of Thiazole Derivatives ***4a**–**f*** and ***7a**–**d***

#### 3.2.1. Method A

A mixture of 1,4-dioxo-3,4-dihydrophthalazine-2(1*H*)-carbothioamide (**1**, 0.221 g, 1 mmol) and the appropriate hydrazonoyl halides **2a**–**f** or **5a**–**d** (1 mmol) in ethanol (20 mL) containing triethylamine (0.1 g, 1 mmol) was refluxed for 3–6 h (monitored by TLC). The formed precipitate was isolated by filtration, washed with methanol, dried, and recrystallized from the appropriate solvent to give products **4a**–**f** and **7a**–**d**, respectively. The physical properties and spectral data of the obtained products are listed below.

#### 3.2.2. Method B

To a mixture of equimolar amounts of compound **1** (0.221 g, 1 mmol) and the appropriate hydrazonoyl halides **2a**–**f** or **5a**–**d** (1 mmol) in ethanol (20 mL), was added TEA (0.1 g). The reaction mixture was irradiated by an ultrasonic generator in a water bath at 50 °C for 30–60 min (irradiation was continued till all of the starting materials had disappeared and the product was formed, as monitored by TLC). The solid that formed after cooling was filtered and recrystallized from appropriate solvent to give compounds **4a**–**f** and **7a**–**d**, respectively.

*2-(4-Methyl-5-(phenyldiazenyl)thiazol-2-yl)-2,3-dihydrophthalazine-1,4-dione* (**4a**). Red solid; m.p. 213–215 °C (Dioxane); IR (KBr): *v* 3411 (NH), 3049, 2931 (C-H), 1668, 1654 (2C=O), 1598 (C=N) cm^−1^; ^1^H-NMR (DMSO-*d*_6_): *δ* 2.55 (s, 3H, CH_3_), 7.02–7.91 (m, 9H, Ar-H), 10.42 (s, br, 1H, NH, D_2_O-exchangeable); ^13^C-NMR (DMSO-*d*_6_): *δ* 16.1, 106.1, 114.3, 115.5, 115.7, 126.4, 128.4, 129.6, 132.5, 146.2, 155.8, 155.9, 160.2, 162.0, 162.5, 165.0. MS *m*/*z* (%): 363 (M^+^). Anal. Calcd. for C_18_H_13_N_5_O_2_S (363.08): C, 59.49; H, 3.61; N, 19.27. Found: C, 59.42; H, 3.58; N, 19.14%.

*2-(4-Methyl-5-(p-tolyldiazenyl)thiazol-2-yl)-2,3-dihydrophthalazine-1,4-dione* (**4b**). Red solid; m.p. 187–189 °C (EtOH); IR (KBr): *v* 3423 (NH), 3027, 2918 (C-H), 1670, 1654 (2C=O), 1603 (C=N) cm^−1^; ^1^H-NMR (DMSO-*d*_6_): *δ* 2.27 (s, 3H, CH_3_), 2.57 (s, 3H, CH_3_), 7.10–8.06 (m, 8H, Ar-H), 9.18 (s, br, 1H, NH, D_2_O-exchangeable); MS *m*/*z* (%): 377 (M^+^). Anal. Calcd. for C_19_H_15_N_5_O_2_S (377.09): C, 60.46; H, 4.01; N, 18.56. Found: C, 60.39; H, 4.07; N, 18.44%.

*2-(4-Methyl-5-(o-tolyldiazenyl)thiazol-2-yl)-2,3-dihydrophthalazine-1,4-dione* (**4c**). Red solid; m.p. 206–208 °C (EtOH); IR (KBr): *v* 3426 (NH), 3031, 2921 (C-H), 1667, 1559 (2C=O), 1590 (C=N) cm^−1^; ^1^H-NMR (DMSO-*d*_6_): *δ* 2.32 (s, 3H, CH_3_), 2.60 (s, 3H, CH_3_), 7.18–7.94 (m, 8H, Ar-H), 10.47 (s, br, 1H, NH, D_2_O-exchangeable); MS *m*/*z* (%): 377 (M^+^). Anal. Calcd. for C_19_H_15_N_5_O_2_S (377.09): C, 60.46; H, 4.01; N, 18.56. Found: C, 60.42; H, 4.00; N, 18.47%.

*2-(5-((4-Methoxyphenyl)diazenyl)-4-methylthiazol-2-yl)-2,3-dihydrophthalazine-1,4-dione* (**4d**). Red solid; m.p. 177–178 °C (EtOH); IR (KBr): *v* 3429 (NH), 3029, 2922 (C-H), 1683, 1650 (2C=O), 1598 (C=N) cm^−1^; ^1^H-NMR (DMSO-*d*_6_): *δ* 2.57 (s, 3H, CH_3_), 3.80 (s, 3H, OCH_3_), 6.93–8.05 (m, 8H, Ar-H), 10.27 (s, br, 1H, NH, D_2_O-exchangeable); MS *m*/*z* (%): 393 (M^+^). Anal. Calcd. for C_19_H_15_N_5_O_3_S (393.09): C, 58.01; H, 3.84; N, 17.80. Found: C, 57.87; H, 3.75; N, 17.69%.

*2-(5-((4-Chlorophenyl)diazenyl)-4-methylthiazol-2-yl)-2,3-dihydrophthalazine-1,4-dione* (**4e**). Red solid; m.p. 237–239 °C (DMF); IR (KBr): *v* 3423 (NH), 3042, 2924 (C-H), 1671, 1640 (2C=O), 1591 (C=N) cm^−1^; ^1^H-NMR (DMSO-*d*_6_): *δ* 2.58 (s, 3H, CH_3_), 7.23–7.89 (m, 8H, Ar-H), 10.04 (s, br, 1H, NH, D_2_O-exchangeable); MS *m*/*z* (%): 397 (M^+^). Anal. Calcd. for C_18_H_12_ClN_5_O_2_S (397.04): C, 54.34; H, 3.04; N, 17.60. Found: C, 54.24; H, 3.05; N, 17.47%.

*2-(4-Methyl-5-((4-nitrophenyl)diazenyl)thiazol-2-yl)-2,3-dihydrophthalazine-1,4-dione* (**4f**). Red solid; m.p. 225–227 °C (DMF); IR (KBr): *v* 3425 (NH), 3042, 2925 (C-H), 1673, 1655 (2C=O), 1594 (C=N) cm^−1^; ^1^H-NMR (DMSO-*d*_6_): *δ* 2.57 (s, 3H, CH_3_), 7.38–8.20 (m, 8H, Ar-H), 10.71 (s, br, 1H, NH, D_2_O-exchangeable); MS *m*/*z* (%): 408 (M^+^). Anal. Calcd. for C_18_H_12_N_6_O_4_S (408.06): C, 52.94; H, 2.96; N, 20.58. Found: C, 52.80; H, 2.90; N, 20.49%.

*2-(4-Oxo-5-(2-phenylhydrazono)-4,5-dihydrothiazol-2-yl)-2,3-dihydrophthalazine-1,4-dione* (**7a**). Yellow solid; m.p. 168–170 °C (EtOH); IR (KBr): *v* 3371, 3178 (2NH), 3051, 2978 (C-H), 1705, 1644, 1621 (3C=O), 1600 (C=N) cm^−1^; ^1^H-NMR (DMSO-*d*_6_): *δ* 6.94–7.96 (m, 9H, Ar-H), 8.63, 10.64 (2s, br, 2H, 2NH, D_2_O-exchangeable); ^13^C-NMR (DMSO-*d*_6_): *δ* 121.5, 126.8, 128.8, 129.3, 130.5, 132.1, 136.4, 138.1, 143.3, 147.4, 149.2, 151.5, 162.2, 164.7, 188.1. MS *m*/*z* (%): 365 (M^+^). Anal. Calcd. for C_17_H_11_N_5_O_3_S (365.06): C, 55.88; H, 3.03; N, 19.17. Found: C, 55.73; H, 3.02; N, 19.11%.

*2-(4-Oxo-5-(2-(p-tolyl)hydrazono)-4,5-dihydrothiazol-2-yl)-2,3-dihydrophthalazine-1,4-dione* (**7b**). Yellow solid; m.p. 179–181 °C (EtOH); IR (KBr): *v* 3426, 3178 (2NH), 3030, 2922 (C-H), 1703, 1649, 1632 (3C=O), 1601 (C=N) cm^−1^; ^1^H-NMR (DMSO-*d*_6_): *δ* 2.26 (s, 3H, CH_3_), 6.99–7.97 (m, 8H, Ar-H), 10.54, 10.79 (2s, br, 2H, 2NH, D_2_O-exchangeable); MS *m*/*z* (%): 379 (M^+^). Anal. Calcd. for C_18_H_13_N_5_O_3_S (379.07): C, 56.98; H, 3.45; N,18.46. Found: C, 56.75; H, 3.40; N, 18.36%.

*2-(4-Oxo-5-(2-(o-tolyl)hydrazono)-4,5-dihydrothiazol-2-yl)-2,3-dihydrophthalazine-1,4-dione* (**7c**). Yellow solid; m.p. 157–159 °C (EtOH); IR (KBr): *v* 3428, 3179 (NH), 3049, 2925 (C-H), 1738, 1650, 1627 (3C=O), 1598 (C=N) cm^−1^; ^1^H-NMR (DMSO-*d*_6_): *δ* 2.17 (s, 3H, CH_3_), 6.97–7.93 (m, 8H, Ar-H), 9.93, 10.25 (2s, br, 2H, 2NH, D_2_O-exchangeable); MS *m*/*z* (%): 379 (M^+^). Anal. Calcd. for C_18_H_13_N_5_O_3_S (379.07): C, 56.98; H, 3.45; N, 18.46. Found: C, 56.84; H, 3.40; N, 18.35%.

*2-(5-(2-(4-Chlorophenyl)hydrazono)-4-oxo-4,5-dihydrothiazol-2-yl)-2,3-dihydrophthalazine-1,4-dione* (**7d**). Yellow solid; m.p. 185–187 °C (EtOH/DMF); IR (KBr): *v* 3429, 3183 (NH), 3054, 2926 (C-H), 1735, 1657, 1629 (3C=O), 1599 (C=N) cm^−1^; ^1^H-NMR (DMSO-*d*_6_): *δ* 7.33–7.97 (m, 8H, Ar-H), 10.27, 10.83 (2s, br, 2H, 2NH, D_2_O-exchangeable); MS *m*/*z* (%): 399 (M^+^). Anal. Calcd. For C_17_H_10_ClN_5_O_3_S (399.02): C, 51.07; H, 2.52; N, 17.52. Found: C, 51.01; H, 2.39; N, 17.42%.

### 3.3. Synthesis of Thiazole Derivatives ***12**–**15***

#### 3.3.1. Method A

A mixture of 1,4-dioxo-3,4-dihydrophthalazine-2(1*H*)-carbothioamide (**1**, 0.221 g, 1 mmol) and α-bromoketones **8**–**11** (1 mmol) in ethanol (20 mL) was refluxed for 4–6 h (monitored by TLC). The product started to separate out during the course of reaction. The solid product was filtered, washed with water, dried and recrystallized from appropriate solvent to give the corresponding thiazoles **12**–**15**, respectively.

#### 3.3.2. Method B

A equimolar mixture of compound **1** (0.221 g, 1 mmol) and the appropriate and α-bromoketones **8**–**11** (1 mmol) in ethanol (20 mL) was irradiated by an ultrasonic generator in a water bath at 50 °C for 30–60 min (irradiation was continued until all of the starting materials disappeared and the product was formed, as monitored by TLC). The solid that formed after cooling was filtered and recrystallized from an appropriate solvent to give the corresponding thiazoles **12**–**15**, respectively.

*2-(4-(4-Nitrophenyl)thiazol-2-yl)-2,3-dihydrophthalazine-1,4-dione* (**12**). Yellow solid; m.p. 193–195 °C (EtOH); IR (KBr): *v* 3430 (NH), 3047, 2922 (C-H), 1658, 1643 (2C=O), 1597 (C=N) cm^−1^; ^1^H-NMR (DMSO-*d*_6_): *δ* 6.98–8.38 (m, 9H, Ar-H and thiazole-H5), 8.81 (s, br, 1H, NH, D_2_O-exchangeable); ^13^C-NMR (DMSO-*d*_6_): *δ* 112.4, 116.1, 125.8, 127.9, 129.3, 129.6, 130.3, 133.0, 134.7, 138.5, 145.3, 148.4, 149.9, 160.6, 163.0. MS *m*/*z* (%): 366 (M^+^). Anal. Calcd. for C_17_H_10_N_4_O_4_S (366.04): C, 55.73; H, 2.75; N, 15.29. Found: C, 55.59; H, 2.72; N, 15.15%.

*2-(4-(5-Methyl-1-phenyl-1H-pyrazol-4-yl)thiazol-2-yl)-2,3-dihydrophthalazine-1,4-dione* (**13**). Yellow solid; m.p. 230–232 °C (dioxane); IR (KBr): *v* 3425 (NH), 3040, 2925 (C-H), 1673, 1649 (2C=O), 1596 (C=N) cm^−1^; ^1^H-NMR (DMSO-*d*_6_): *δ* 2.59 (s, 3H, CH_3_), 7.46–7.62 (m, 6H, Ar-H and thiazole-H5), 7.78 (s, 1H, pyrazole-H3), 7.91–8.15 (m, 4H, Ar-H), 9.88 (s, br, 1H, NH, D_2_O-exchangeable); MS *m*/*z* (%): 401 (M^+^). Anal. Calcd. For C_21_H_15_N_5_O_2_S (401.09): C, 62.83; H, 3.77; N, 17.45. Found: C, 62.77; H, 3.64; N, 17.38%.

*2-(4-(Pyridin-2-yl)thiazol-2-yl)-2,3-dihydrophthalazine-1,4-dione* (**14**). Yellow solid; m.p. 200–202 °C (EtOH); IR (KBr): *v* 3427 (NH), 3048, 2924 (C-H), 1666, 1643 (2C=O), 1589 (C=N) cm^−1^; ^1^H-NMR (DMSO-*d*_6_): *δ* 7.23–8.65 (m, 9H, Ar-H), 8.90 (s, br, 1H, NH, D_2_O-exchangeable); ^13^C-NMR (DMSO-*d*_6_): *δ* 106.5, 115.5, 115.7, 125.2, 126.2, 126.3, 129.8, 131.9, 132.6, 135.2, 146.6, 155.7, 156.1, 159.4, 162.6, 165.1. MS *m*/*z* (%): 322 (M^+^). Anal. Calcd. for C_16_H_10_N_4_O_2_S (322.05): C, 59.62; H, 3.13; N, 17.38. Found: C, 59.51; H, 3.10; N, 17.25%.

*2-(4-(2-Oxo-2H-chromen-3-yl)thiazol-2-yl)-2,3-dihydrophthalazine-1,4-dione* (**15**). Yellow solid; m.p. 253–255 °C (Dioxane); IR (KBr): *v* 3429 (NH), 3032, 2928 (C-H), 1721, 1670, 1656 (3C=O), 1606 (C=N) cm^−1^; ^1^H-NMR (DMSO-*d*_6_): *δ* 7.37–8.65 (m, 8H, Ar-H), 8.36 (s, 1H, coumarine-H4), 8.64 (s, 1H, thiazole-H5), 8.87 (s, br, 1H, NH, D_2_O-exchangeable); MS *m*/*z* (%): 389 (M^+^). Anal. Calcd. for C_20_H_11_N_3_O_4_S (389.05): C, 61.69; H, 2.85; N, 10.79. Found: C, 61.64; H, 2.79; N, 10.58%.

### 3.4. Synthesis of Bis-thiazole Derivatives ***18*** and ***19***

#### 3.4.1. Method A

A mixture of 1,4-dioxo-3,4-dihydrophthalazine-2(1*H*)-carbothioamide (**1**, 0.221 g, 1 mmol) and *bis*-α-bromoketones **16** and compound **17** (2 mmol) in ethanol (20 mL) was refluxed for 4–7 h (monitored by TLC). The product started to separate out during the course of the reaction. The solid product was filtered, washed with water, dried and recrystallized from DMF to give the corresponding *bis*-thiazoles **18** and **19**, respectively.

#### 3.4.2. Method B

A mixture of 1,4-dioxo-3,4-dihydrophthalazine-2(1*H*)-carbothioamide (**1**, 0.221 g, 1 mmol) and *bis*-α-bromoketones **16** and compound **17** (2 mmol) in ethanol (20 mL) was irradiated by an ultrasonic generator in a water bath at 50 °C for 30–60 min. (irradiation was continued until all of the starting materials have been disappeared and the product was formed, monitored by TLC). The solid that formed after cooling was filtered and recrystallized from DMF to give the corresponding bisthiazoles **18** and **19**, respectively.

*2,2′-(4,4′-(1,4-Phenylene)bis(thiazole-4,2-diyl))bis(2,3-dihydrophthalazine-1,4-dione)* (**18**). Yellow solid; m.p. 309–311 °C; IR (KBr): *v* 3427 (NH), 3039, 2924 (C-H), 1669, 1648 (2C=O), 1610 (C=N) cm^−1^; ^1^H-NMR (DMSO-*d*_6_): *δ* 7.11–8.02 (m, 12H, Ar-H), 8.11 (s, 2H, thiazole-H5), 8.87 (s, br, 2H, 2NH, D_2_O-exchangeable); ^13^C-NMR (DMSO-*d*_6_): *δ* 112.8, 116.0, 119.2, 125.8, 128.4, 129.5, 130.8, 131.1, 135.3, 143.8, 148.8, 160.4, 162.8. MS *m*/*z* (%): 564 (M^+^). Anal. Calcd. for C_28_H_16_N_6_O_4_S_2_ (564.07): C, 59.56; H, 2.86; N, 14.89. Found: C, 59.69; H, 2.74; N, 14.75%.

*2,2'-(4,4'-(3,4-Dimethylthieno[2,3-b]thiophene-2,5-diyl)bis(thiazole-4,2-diyl))bis(2,3-dihydrophthalazine-1,4-dione)* (**19**). Brown solid; m.p. 276–278 °C; IR (KBr): *v* 3429 (NH), 3062, 2921 (C-H), 1670, 1652 (2C=O), 1601 (C=N) cm^−1^; ^1^H-NMR (DMSO-*d*_6_): *δ* 2.37 (s, 6H, 2CH_3_), 7.06–8.07 (m, 8H, Ar-H), 8.38 (s, 2H, thiazole-H5), 9.23 (s, br, 2H, 2NH, D_2_O-exchangeable); MS *m*/*z* (%): 654 (M^+^). Anal. Calcd. for C_30_H_18_N_6_O_4_S_4_ (654.03): C, 55.03; H, 2.77; N, 12.84. Found: C, 54.86; H, 2.61; N, 12.73%.

### 3.5. Agar Diffusion Medium Biological Assays

All compounds were screened in vitro for their antimicrobial activity, applying an agar diffusion method [34]. A suspension of the organisms was added to sterile nutrient agar media at 45 °C and the mixture was transferred to sterile Petri dishes and allowed to solidify. Holes of 6 mm in diameter were made using a cork borer. The samples of the test compounds and of reference drugs, were dissolved in DMSO to give a solution of 5 mg·mL^−1^. The amount tested of either the synthesized compounds or reference drugs was 100 µL. Dimethylsulfoxide (DMSO) was used as a negative control. The plates were left for 1h at room temperature as a period of pre-incubation diffusion to minimize the effects of variation in time between the applications of the different solutions. The plates were then incubated at 37 °C for 24 h and observed for antimicrobial activity. The diameters of inhibition zone were measured and compared with those of the reference drugs. The observed inhibition zones were measured in millimeters beyond good diameters. Also, the percentage values of inhibition zones as compared to reference drugs were recorded (Table 4).

## 4. Conclusions

In summary, we have developed a new green methodology and synthesized several novel 2-thiazolylphthalazine derivatives using ultrasound irradiation, resulting in high and efficient yields in short reaction times. The antimicrobial activities of all prepared compounds were evaluated and the results revealed the promising activities of compounds **4d**, **7b** (117% zone inhibition), and **7c** (105% zone inhibition) against *Salmonella sp.*, exceeding the inhibition zones of the reference drug Gentamycin.

## Figures and Tables

**Table 1:** Optimization of the synthesis of 2-(4-methyl-5-(phenyl-diazenyl)thiazol-2-yl)-2,3-dihydro- phthalazine-1,4-dione (**4a**) under ultrasonic irradiation.

Entry	Solvent	Temperature (°C)	Time (min)	Yield (%)
1	EtOH	r.t.	40	90
2	EtOH	50	35	92
3	Dioxane	r.t.	60	82
4	Dioxane	50	45	87
5	DMF	r.t.	40	85
6	DMF	50	35	85
7	Benzene	r.t.	45	89
8	Benzene	50	40	91
9	Toluene	r.t.	60	87
10	Toluene	50	50	90

**Table 2:** Comparison of synthesis of thiazolylphthalazines **4** and **7** under ultrasound irradiation and conventional heating.

Product No.	Conventional		Sonication	
	Time (h)	Yield (%)	Time (min)	Yield (%)
**4a**	4	68	35	92
**4b**	4	69	50	84
**4c**	6	70	50	88
**4d**	4	71	40	86
**4e**	4	74	55	88
**4f**	3	67	45	87
**7a**	6	65	60	85
**7b**	5	67	40	83
**7c**	5	68	50	85
**7d**	4	69	55	83

**Table 3:** Comparison of synthesis of thiazolylphthalazines **12**–**15** and **18**, **19** under ultrasound irradiation and conventional heating.

Product No.	Conventional		Sonication	
	Time (h)	Yield (%)	Time (min)	Yield (%)
**12**	5	70	35	84
**13**	4	67	50	82
**14**	4	72	60	88
**15**	6	69	40	84
**18**	5	70	55	85
**19**	7	68	45	83

**Table 4:** Antimicrobial activity of the new compounds compared to reference drug.

Compd. No. ^a^	Inhibition Zone Diameter in mm and (%) Value					
	Gram-Positive Bacteria		Gram-Negative Bacteria		Fungi	
	SA	BS	S SP.	EC	AF	CA
**1**	NA	12 (37%)	16 (94%)	NA	8 (34%)	13 (52%)
**4a**	8 (34%)	11 (34%)	15 (88%)	11 (57%)	NA	11 (44%)
**4b**	16 (69%)	16 (50%)	15 (88%)	16 (84%)	10 (43%)	NA
**4c**	11 (47%)	13 (40%)	14 (82%)	13 (68%)	12 (52%)	15 (60%)
**4d**	10 (43%)	13 (40%)	20 (117%)	10 (52%)	NA	13 (52%)
**4e**	12 (52%)	NA	9 (52%)	NA	NA	12 (48%)
**4f**	14 (60%)	12 (37%)	15 (88%)	15 (78%)	12 (52%)	15 (60%)
**7a**	NA	10 (31%)	NA	NA	NA	10 (40%)
**7b**	NA	9 (28%)	20 (117%)	9 (47%)	8 (34%)	12 (48%)
**7c**	NA	12 (37%)	18 (105%)	10 (52%)	9 (39%)	14 (56%)
**7d**	NA	NA	NA	NA	NA	NA
**12**	NA	11 (34%)	NA	NA	NA	11 (44%)
**13**	NA	12 (37%)	NA	NA	NA	NA
**14**	12 (52%)	NA	12 (70%)	16 (84%)	14 (60%)	NA
**15**	14 (60%)	12 (37%)	9 (52%)	9 (47%)	16 (69%)	NA
**18**	15 (65%)	15 (46%)	15 (88%)	15 (78%)	15 (65%)	21 (84%)
**19**	11 (47%)	11 (34%)	10 (58%)	NA	8 (34%)	15 (60%)
AmphotericinB	-	-	-	-	23 (100%)	25 (100%)
Ampicillin	23 (100%)	32 (100%)	-	-	-	-
Gentamycin	-	-	17 (100%)	19 (100%)	-	-

^a^ conc.=5 mg·mL^−1^, negative control (DMSO). NA (indicates no activity). Tested fungi: *Aspergillus fumigates* (AF) and *Candida albicans (*CA). Tested gram-positive bacteria: *Staphylococcus aureus* (SA) and *Bacillus subtilis* (BS). Tested gram-negative bacteria: *Salmonella sp*. (S SP.), *Esherichia coli* (EC). The experiment was carried out in triplicate and the values represent the average zone of inhibition.
